# Patterns of Diversity of *Fusarium* Fungi Contaminating Soybean Grains

**DOI:** 10.3390/toxins13120884

**Published:** 2021-12-10

**Authors:** Maciej Żelechowski, Tomasz Molcan, Katarzyna Bilska, Kamil Myszczyński, Jacek Olszewski, Krzysztof Karpiesiuk, Joanna Wyrębek, Tomasz Kulik

**Affiliations:** 1 Department of Botany and Nature Protection, University of Warmia and Mazury in Olsztyn, Plac Łódzki 1, 10-727 Olsztyn, Poland; katarzyna.bilska@uwm.edu.pl (K.B.); joanna.wyrebek@uwm.edu.pl (J.W.); 2 Department of Bioinformatics, Institute of Biochemistry and Biophysics, Polish Academy of Sciences, Adolfa Pawińskiego 5A, 02-106 Warsaw, Poland; tomasz.molcan@gmail.com; 3 Molecular Biology Laboratory, Institute of Animal Reproduction and Food Research, Polish Academy of Sciences, 10-748 Olsztyn, Poland; kamil.myszczynski@gmail.com; 4 Experimental Education Unit, Oczapowskiego 8, 10-719 Olsztyn, Poland; jacolsz@uwm.edu.pl; 5 Department of Pig Breeding, University of Warmia and Mazury in Olsztyn, ul. Oczapowskiego 5, 10-719 Olsztyn, Poland; krzysztof.karpiesiuk@moskit.uwm.edu.pl

**Keywords:** *Fusarium*, *F. avenaceum*, Equiseti clade, phylogenetic analysis, soybean grains

## Abstract

Soybean is an important, high protein source of food and feed. However, like other agricultural grains, soybean may pose a risk to human and animal health due to contamination of the grains with toxigenic Fusaria and associated mycotoxins. In this study, we investigated the diversity of Fusaria on a panel of 104 field isolates obtained from soybean grains during the growing seasons in 2017–2020. The results of species-specific PCR analyses showed that *Fusarium avenaceum* was the most common (*n* = 40) species associated with soybean grains in Poland, followed by *F. equiseti* (*n* = 22) and *F. sporotrichioides* (11 isolates). A set of isolates, which was not determined based on PCR analyses, was whole genome sequenced. Multiple sequence analyses using *tef-1α*, *top1*, *rpb1*, *rpb2*, *tub2, pgk, cam* and *lsu* genes showed that most of them belonged to Equiseti clade. Three cryptic species from this clade: *F. clavum, F. flagelliforme* and FIESC 31 (lacking Latin binomial) were found on soybean for the first time. This is the first report demonstrating the prevalence of Fusaria on soybean grains in Poland.

## 1. Introduction

The continuous growth of the global population demands an improvement of protein production with an environmentally friendly and energy-efficient practice. The integration of protein-rich legumes into cropping systems appears to be among the most promising strategies to bridge the gap between global food and feed demand and supply. Soybean is one of the most important crops worldwide with the highest protein content (40–42%) of all crops and is the second, after groundnut, to oil content (18–22%) of legumes [1,2]. It is currently the most widely cultivated legume crop occupying around 6% of the total land surface [3]. However, soybean production is threatened by a variety of pathogens [4,5]. Among the most economically important are fungi belonging to the Fusarium solani species complex responsible for soybean sudden death syndrome [6] and the Fusarium oxysporum species complex causing soybean root rot and seedling blight [7].

In addition, a range of other Fusaria such as *F. verticillioides* (Sacc.) Nirenberg [8], *F. sporotrichioides* Scherb [9]*, F. equiseti* (Corda) Sacc. [10]*, F. semitectum* Berk. and Ravenel [11], *F. fujikuroi* Nirenberg [8]*, F. graminearum* Schwabe [12]*, F. proliferatum* (Matsush.) Nirenberg [8] and fungi from F. incarnatum-equiseti species complex may be involved in the contamination of soybean grains posing threat to human and animal health due to mycotoxin production [13,14,15,16,17,18,19] (Table 1).

However, it is worth noting that, in contrast to other grains such as wheat [20], barley [21] or corn [22], knowledge of *Fusarium* fungi and associated mycotoxins on soybean grains is scarce (Table 1).

To fill this gap, we studied the diversity of this group of toxigenic fungi on a panel of 104 field isolates recovered from soybean grains during the 2017–2020 growing seasons. Contrary to previous studies, our results highlight the predominance of enniatin genotypes of *F. avenaceum* in Polish soybean grains. We also showed that nearly one-fifth of isolates tested by species-specific assays did not give any positive results preventing their identification. Therefore, whole-genome sequencing was performed to clarify their taxonomic status. Multiple sequence comparisons using *tef-1α*, *top1*, *rpb1*, *rpb2*, *tub2, pgk, cam* and *lsu* genes showed that most of them belonged to Equiseti clade. Newly assembled genomes provide great scope for comparative genomics and characterization of mycotoxin gene clusters. This issue will be addressed in a future study.

## 2. Results

### 2.1. Identification of Fusaria by Species-Specific PCR Assays

The plating of diseased soybean grains on PDA plates allowed to isolate a total of 104 *Fusarium*-like colonies, which were then subjected to molecular analyses (Table S1). PCR analyses using species-specific primers allowed determining 80 isolates to the species level. Forty isolates were identified as *F. avenaceum* (Fr.) Sacc. [10]*,* 22 isolates as *F. equiseti*, 11 isolates as *F. sporotrichioides,* six isolates as *F. graminearum* and one isolate as *F. culmorum* (Wm.G. Sm.) Sacc. [23]. Each isolate of *F. avenaceum* gave a positive result with the assay determining *esyn1* genotype.

### 2.2. Identification of Fusaria through Sequence Comparisons

Nineteen isolates that did not give positive signals with qPCR as well as five isolates from the 2020 growing season (which were not subjected to PCR) were whole genome sequenced. For the purpose of sequence comparison, an additional 16 isolates that were identified using PCR were also sequenced. To determine their taxonomic affiliation, we performed BLASTn searches against the NCBI database using eight genes: *tef-1α* (translation elongation factor 1 alpha), *top1* (topoisomerase 1), *tub2* (tubulin beta chain), *pgk* (phosphoglycerate kinase), *rpb1* (DNA-directed RNA polymerase II largest subunit), and *rpb2* (DNA-directed RNA polymerase II second largest subunit), *cam* (calmodulin) and *lsu* (large-subunit rRNA gene) genes. Selected genes have been previously shown to resolve phylogenetic relationships of diverse Fusaria [24,25,26]. The results of BLAST searches are shown in Table S2. Twenty-one isolates were determined to belong to Equiseti clade, 5 isolates were identified as *F. avenaceum*, five isolates as *F. oxysporum*, one as *F. sporotrichioides* and one as *F. cerealis* (Cooke) Sacc [10].

Assuming > 99% identity match and ≥75% query coverage, *tef-1α* was the most effective in identifying phylogenetic species from Equiseti clade (Table S2). It is worth noting, however, that the GenBank database provides an informal classification system based on a haplotype nomenclature. In addition, most GenBank entries are assigned a single latin binomial *F. equiseti*, which refers to the morphological species concept (morphospecies). In most cases, BLAST searches using other genes did not allow resolving taxonomic issues in this clade mostly due to the lack of reference sequences in the GenBank database. *Tef-1α*-based analysis showed that, among 21 isolates from Equiseti clade, 12 were determined as *F. equiseti*, six as *F. flagelliforme* (J.W. Xia, L. Lombard, Sand.-Den., X.G. Zhang and Crous) [27]*,* two as FIESC 31 (lacking latin binomial) (J.W. Xia, L. Lombard, Sand.-Den., X.G. Zhang and Crous) [27] and one as *F. clavum* (J.W. Xia, L. Lombard, Sand.-Den., X.G. Zhang and Crous) [27].

To determine trichothecene genotypes of *F. cerealis*, *F. culmorum* and *F. graminearum*, we performed sequence comparisons against the ToxGen database [28] using complete sequence of Tri12 gene. Results of analyses showed that both *F. cerealis* (S18/34) and *F. culmorum* (S18/1) yielded 100% sequence identity to NIV genotypes: AY102569 and KU572425, respectively. An isolate S18/4 of *F. graminearum* yielded 100% sequence identity to 3ADON genotype (KU572434), while the remaining three isolates S18/49, S18/55 and S18/66 had the highest identity to the 15ADON genotype (HG970333).

### 2.3. Phylogenetic Analysis

Phylogenetic analyses were performed using nucleotide sequences of *tef-1α*, *top1*, *rpb1*, *rpb2*, *tub2, pgk, cam* and *lsu* genes. Estimates of genetic diversity (indels, SNPs, nucleotide diversity values and the percent of polymorphic sites) are provided in Table 2.

The phylogenetic relationships among isolates were inferred using Bayesian inference (BI). Strains were resolved into two main sister clades by nucleotide variations within the sequence of *tef-1α.* The first clade included isolates of *F. clavum, F. flagelliforme* and FIESC 31 in three species specific clades, while the second sister clade included all *F. equiseti* isolates (Figure S1). Similar topologies were also found with phylogenetic trees for *rpb1* (Figure S2), *rpb2* (Figure S3) and *cam* (Figure S4).

A tree based on *tub2* sequences showed a slightly different topology and showed a closer relationship of *F. clavum* to *F. equiseti* compared to the remaining two species (Figure S5). A similar finding was also evident for *top1* by clustering *F. clavum* (S19/5) into the second sister clade together with all *F. equiseti* isolates (Figure S6). Phylogenetic analysis of *pgk* sequences showed contrasting results and grouped *F. clavum* into a well-supported clade together with isolates of FIESC 31 (Figure S7). The *lsu* tree failed to resolve strains of *F. clavum* and FIESC 31*,* presumably due to the low number of SNPs (Figure S8, Table 2).

**Figure 1:**
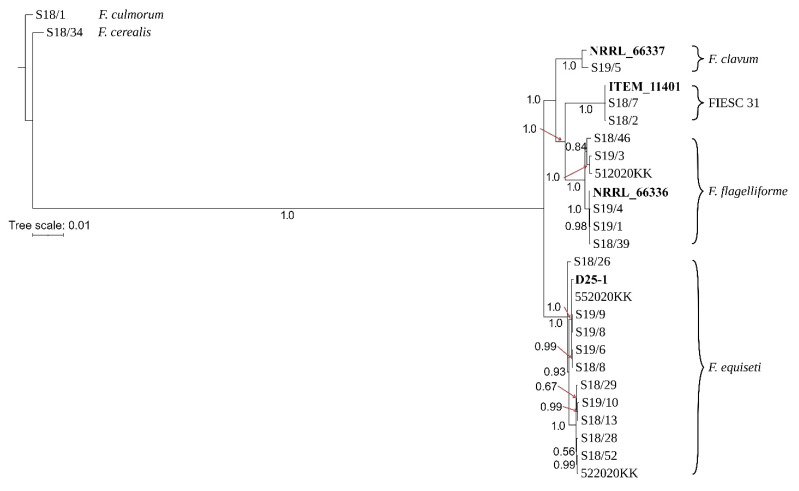
The phylogenetic tree resulting from a Bayesian analysis on the combined alignment of eight loci (*tef-1α*, *top1*, *rpb1*, *rpb2*, *tub2, pgk, cam* and *lsu*) for *Fusarium* spp. Bayesian posterior probability scores are shown at the nodes. The scale bar represents the expected number of changes per site. The reference strains are indicated in bold. The tree was rooted to *F. culmorum* S 18/1.

The differences in phylogenetic relationships among these cryptic species could be explained by incomplete lineage sorting or more recent inter-species gene exchange. The impact of incomplete lineage sorting and recombination on the evolution of Equiseti clade could also be observed on *top1*, which failed to group all strains of *F. flagelliforme* into a species-specific clade (Figure S6). Phylogenetic analysis of *pgk* sequences showed a different topology than the remaining trees and placed *F. flagelliforme* isolates into divergent clades occupying the basal position in the phylogenetic tree (Figure S7). A combined phylogenetic tree provided similar topology to *tef-1α*, *rpb1*, *rpb2* and *cam* trees, and grouped all isolates into four well-supported species-specific clades (Figure 1).

## 3. Discussion

The knowledge of fungal patterns contaminating crops is fundamental for understanding the population ecology, dynamics and evolutionary relationships of fungi [29]. Soybean grains may be contaminated by a range of Fusaria [13,14,15,16,17,18,19] (Table 1). However, contrary to previous studies, our results highlight the predominance of *F. avenaceum*, which, to date, was rarely reported on soybean [30]. It is worth noting that the high prevalence of *F. avenaceum* in tested isolates is in line with our previous study on other protein-rich crops, such as common vetch, faba bean and blue lupine [31]. In small-grain cereals, *F. avenaceum* appears to be more commonly responsible for the crown rot and head blight that negatively results in yield and quality of grain [32]. *F. avenaceum* was recently detected during FHB epidemics in Poland, although with far less frequency than *F. graminearum* [33].

In this study, 37 isolates recovered from soybean were determined to belong to the Equiseti clade. This clade, together with the Incarnatum clade, forms the FIESC complex involving 33 phylogenetically distinct species, which can be resolved based on Multi-Locus Sequence Typing (MLST) [34,35,36]. Members from both Equiseti and Incarnatum clades are mainly associated with crops and soil [37]. Several reports have documented a prevalence of fungi from the FIESC complex on soybean [14,18,19,37]. However, for some (especially older) reports, it is impossible to gather information on the cryptic diversity within the FIESC due to the fact that a number of species have been previously treated as synonyms of *F. equiseti*. A more recent MLST-based characterization of the FIESC complex showed that *F. ipomoeae* (M.M. Wang, Qian Chen and L. Cai) [36]*, F. sulawesiense* (Maryani, Sand.-Den., L. Lombard, Kema and Crous) [38] and *F. luffae* (M.M. Wang, Qian Chen and L. Cai) [36] are mainly associated with soybean in China [19]. Surveys from Ethiopia and Ghana showed that most of the isolates recovered from soybean roots represented novel, undescribed species [37]. The complex nature of FIESC from soybean was also highlighted in this study. We showed that four species from the Equiseti clade are responsible for the contamination of soybean grains; however, with variable species richness patterns. No members from Incarnatum clade were detected. Among the 21 isolates subjected to whole-genome sequencing, more than half were determined as *F. equiseti*. This cryptic species appears to be broadly distributed in agroecosystems. To date, the vast majority of characterized *F. equiseti* strains were recovered from either plant material or soil/sediment substrates [27]. Six isolates recovered in this study were identified as *F. flagelliforme*. This cryptic species appears to be restricted to Europe, and according to our knowledge, there are no reports showing the incidence of this species on hosts other than cereals [27]. Two remaining species, *F. clavum* and FIESC 31, were also found to be associated with soybean for the first time. The broad distribution of *F. clavum* was recently indicated by screening a number of isolates recovered from environmental, plant and human samples in Africa, Asia, Europe and North America [27,34]. Knowledge on the geographic distribution of FIESC 31 is scarce. To date, only two strains of this cryptic species have been described [39].

Fusaria are well known as producers of a vast array of mycotoxins such as enniatins, trichothecenes, fumonisins and zearalenone, which are frequently found in grains and processed foods [40]. They are synthesized through a range of secondary metabolite gene clusters. The distribution of these clusters in fungal genomes is often not correlated with the phylogenetic relationships of species [39,41]. For some fungal lineages, their irregular distribution may also be observed at the strain level [41]. The results presented in this study may indicate potential contamination of soybean with enniatins and moniliformin, which are often found in cereal foods as the result of contamination of the grains with *F. avenaceum* [42]. Enniatins are mainly produced by strains harboring the *esyn1* gene, which was detected in all examined isolates of *F. avenaceum* [32,42]. FIESC members are able to produce diverse mycotoxins, however, the mycotoxin contamination of crops with this fungal complex is unclear [39]. Previous studies by Barros et al. (2014) [43] and Hartman et al. (2019) [37] showed that FIESC isolates obtained from soybean produced a range of mycotoxin compounds from both type A and type B trichothecenes. However, the FIESC complex appears to exhibit remarkable variation in the distribution of SM clusters. In contrast to the trichothecene cluster, which appears to be commonly distributed, clusters responsible for the production of the enniatin, fusarin and zearalenone display mosaic distribution [39]. A more comprehensive understanding of the diversity and origin of SM clusters requires analysis of a larger set of genomes. However, for many cryptic species from the FIESC complex, genome characterization has been largely limited by the absence of genomes in the GenBank database. Our study may provide a valuable genomic resource for such a study. Further studies will address this issue by incorporating a larger set of strains from the Equiseti clade recovered from various cereals. Whole genome comparisons will provide an unprecedented opportunity to study their patterns of diversity and evolution.

## 4. Materials and Methods

### 4.1. Field Isolates

**Figure 2:**
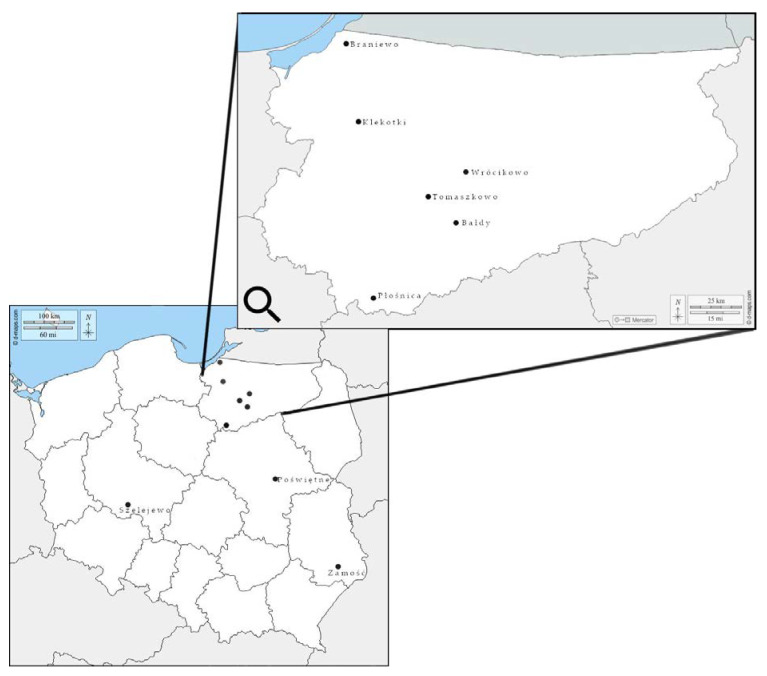
Location of fields in Poland, from which soybean grain were sampled for analyses.

Field isolates were obtained from 17 soybean grain samples (0.5 kg) harvested in 2017–2020 in different regions of Poland (Figure 2). Fifty grains from each sample showing visible symptoms of fungal infection, such as discoloration, black mottling and cracked or shriveled skin, were selected and placed on Petri dishes with distilled water. After 24 h of soaking at room temperature, the grains were surface sterilized with 70% ethanol (EtOH) for 2 min and placed on potato dextrose agar (PDA) (A&A Biotechnology, Gdynia, Poland) in Petri dishes. After 4–6 days of incubation at room temperature in darkness, *Fusarium* resembling colonies were transferred to fresh PDA plates for further molecular analyses. A total of 104 *Fusarium* isolates were assigned with individual strain codes and stored at −25 °C in the fungal collection of the Department of Botany and Nature Protection, University of Warmia and Mazury in Olsztyn, Poland.

### 4.2. DNA Extraction

To obtain genomic DNA, a patch of mycelium (approximately 0.1–0.2 mg) was harvested into homogenization tubes with 1 mm silica spheres (Lysing matrix C, MP Biomedicals, Santa Ana, CA, USA). Homogenization was performed using a FastPrep-24 instrument (MP Biomedicals, Santa Ana, CA, USA). DNA from fungal isolates was extracted with the use of the Genomic Mini AX Food kit according to the manufacturer’s protocol (A&A Biotechnology, Gdynia, Poland).

### 4.3. Identification of Fusarium Species

To ensure recovery of DNA free of amplification inhibitors, FungiQuant assay [44] was first used. Samples with Ct-values (cycle threshold) below 25 were further analyzed with species-specific assays. Each sample was analyzed in three replicates, assuming positive signals of amplification as Ct-values below 30. Besides the identification of species, mycotoxin genotypes were also determined by using various TaqMan assays. We used marker targeting the *esyn1* gene, to determine enniatin genotype for *F. avenaceum* (Table 3).

### 4.4. DNA Sequencing and Assembly

In total, 40 field isolates of *Fusarium* spp. were sequenced by the whole-genome sequencing and included: (I) a group of 19 isolates that could not be determined based on PCR assays, (II) a set of 16 isolates (two isolates per species) that were previously identified to the species level by PCR and (III) 5 isolates isolated in 2020 growing season (which were not included in PCR analyses). Sequencing was conducted by Macrogen (Seoul, South Korea). Libraries were prepared using KAPA HyperPlus Kit (Roche Sequencing Solutions, Pleasanton, CA, USA). An Illumina HiSeq X Ten was used to sequence the genomes using a paired-end read length of 2 × 150 bp with an insert size of 350 bp. The sequencing quality was assessed via FastQC (ver. 0.11.9) [50]. Low-quality reads were trimmed using Trimmomatic (v.0.36) [51] and the genome was assembled via SPAdes (v.3.13.2) [52]. The project was submitted to the NCBI BioProject under accession no: PRJNA730356.

### 4.5. BLAST Analysis

The complete sequences of 6 genes: *tef-1α* (translation elongation factor 1 alpha)*, top1* (topoisomerase I)*, rpb1, rpb2* (RNA polymerase II genes)*, tub2* (beta-tubulin)*, pgk* (phosphoglycinecerate kinase), *cam* (calmodulin) *and lsu* (large-subunit rRNA gene) genes*,* were retrieved from genome sequences with Geneious Prime (v. 2019.0.4 created by Biomatters, Auckland, New Zealand, available from http://www.geneious.com (accessed on 1 November 2021). Identification of the isolates to the species level was done through sequence comparisons using the BLAST searches with default parameters [53]. Species were determined using thresholds of 99–100% nucleotide identity and ≥75% coverage of the query sequence length.

### 4.6. Phylogenetic Analysis

Phylogenetic analyses were performed using *tef-1α, top1, rpb1, rpb2, tub2, pgk, cam and lsu* genes of 21 field isolates from Equiseti clade. In addition, sequence data from strains: D25-1 (*F. equiseti,* GenBank accession no QOHM00000000.1), NRRL 66,337 (*F. clavum,* GenBank accession no QGEC00000000.1), NRRL 66,336 (*F. flagelliforme,* GenBank accession no QHHI00000000) and ITEM 11,401 (FIESC 31, GenBank accession no QHKN00000000.1) was used for comparisons. MAFFT software (v7.453) [54] was used to create sequence alignments.

The best partition schemes and corresponding substitution models for alignment were estimated by means of PartitionFinder2 [55]. Afterwards, based on the alignment and obtained models, Bayesian analysis was conducted using MrBayes 3.2.7 [56]. The Markov chain Monte Carlo (MCMC) algorithm was run for 5,000,000 generations (sampling every 500) with four incrementally heated chains (starting from random trees). The Tracer 1.7.1 [57] software was used to determine the number of generations needed to reach stationarity, which occurred at approximately 500,000 generations. Therefore, the first 1000 trees were discarded as burn-in, and the remaining trees were used to create Bayesian consensus trees. Two strains: *F. cerealis* (S18/34) and *F. culmorum* (S18/1) isolated from soybean grains were used as outgroups.

To reveal nucleotide variation, analyzed genes were extracted and aligned separately using MAFFT software (v.7.453) [54]. Gene polymorphism analyses were conducted for each gene based on the alignment of 24 strains from Equiseti clade. Variation within each gene was identified as a SNP or indel and counted with the use of an in-house Python script. Nucleotide diversity values (*π*) for each gene were calculated with TASSEL software (v.5.2.40) [58]. As nucleotide diversity is based only on nucleotide substitutions, the number of indels and percentage of polymorphic sites are given for each gene.

## Figures and Tables

**Table 1:** *Fusarium* species and associated mycotoxins previously reported on soybean grains.

*Fusarium* Species Reported on Soybean Grains	*Fusarium* Mycotoxins Reported On Soybean Grains	Location, Year of Analysis	References
*F. verticillioides*	fumonisins, type B trichothecenes	Italy, 2008–2010	[13]
*F. sporotrichioides, F. verticillioides, F. equiseti* *, F. semitectum*		Croatia, 2002–2008	[14]
F. graminearum species complex	type B trichothecenes	Argentina, 2012–2014	[15]
	fumonisins, zearalenone, type A and type B trichothecenes	Worldwide sample collection, 2008–2017	[16]
	fumonisins, zearalenone, type A and type B trichothecenes	Nigeria, 2019	[17]
*F. fujikuroi, F. graminearum, F. proliferatum*, F. incarnatum-equiseti species complex		China, 2019	[18]
F. incarnatum-equiseti species complex		China, 2020	[19]

**Table 2:** Variation in *tef-1α, top1, rpb1, rpb2, tub2, pgk, cam and lsu* among isolates from Equiseti clade.

**Gene**	**Length (bp)**	**SNPs**	Indels *	**%PS**	**π**
*tef-1* *α*	727	30	3	4.55	0.03
*top1*	818	17	4	2.57	0.01
*rpb1*	1606	39	0	2.43	0.01
*rpb2*	1853	24	0	1.3	0.01
*tub2*	1352	15	79	6.95	0.04
*pgk*	889	47	2	5.51	0.03
*cam*	712	92	129	31.04	0.05
*lsu*	1074	14	201	20.02	0.05

%PS—percent of polymorphic sites, π—nucleotide diversity values. *—indels include single nucleotide insertions and deletions of longer tracts of DNA.

**Table 3:** List of real-time PCR assays used to determine species and mycotoxin genotypes.

qPCR Assay	Primer/Probe Sequence	Reaction Reagents	Reaction Conditions	References
FungiQuant	GGRAAACTCACCAGGTCCAG	A	95 °C for 20 s, (95 °C for 1 s, 60 °C for 30 s) × 40	[44]
	GSWCTATCCCCAKCACGA			
	Probe:FAM-TGGTGCATGGCCGTT-MGB			
Species				
*F. avenaceum*	CCATCGCCGTGGCTTTC CAAGCCCACAGACACGTTGT Probe: FAM-ACGCAATTGACTATTGC-MGB	B	95 °C for 20 s, (95 °C for 1 s, 60 °C for 50 s) × 40	[45]
*F. culmorum*	TCGTTGACGGTGAGGGTTGT GACTCGAACACGTCAACCAACT Probe:FAM-CGGTTATTATTTCGAAAAGT-MGB	A	95 °C for 20 s, (95 °C for 1 s, 60 °C for 30 s) × 40	[46]
*F. equiseti*	CACCGTCATTGGTATGTTGTCATC TGTTAGCATGAGAAGGTCATGAGTG	C	95 °C for 5 min, (95 °C for 15 s, 65 °C for 60 s) × 40, dissociation curve analysis at 60–95 °C.	[47]
*F. graminearum* s.s.	TGGCCTGAATGAAGGATTTCTAG CATCGTTGTTAACTTATTGGAGATG Probe:FAM-TTAAACACTCAAACACTACA-MGB	A	95 °C for 20 s, (95 °C for 1 s, 60 °C for 30 s) × 40	[48]
*F. langsethiae*	CAAGTCGACCACTGTGAGTACCTCT TGTCAAAGCATGTCAGTAAAGATGAC	C	95 °C for 5 min, (95 °C for 15 s, 65 °C for 60 s) × 40, dissociation curve analysis at 60–95 °C.	[47]
*F. poae*	AAATCGGCGTATAGGGTTGAGATA GCTCACACAGAGTAACCGAAACCT Probe:FAM-CAAAATCACCCAACCGACCCTTTC-TAMRA	B	50 °C for 2 min, 95 °C for 10 min, (95 °C for 15 s, 60 °C for 60 s) × 40	[45]
*F. proliferatum*	CTTCGATCGCGCGTCCT CACGTTTCGAATCGCAAGTG	C	95 °C for 5 min, (95 °C for 15 s, 65 °C for 60 s) × 40, dissociation curve analysis at 60–95 °C.	[47]
*F. sporotrichioides*	GCAAGTCGACCACTGTGAGTACA CTGTCAAAGCATGTCAGTAAAAATGAT	C	95 °C for 5 min, (95 °C for 15 s, 65 °C for 60 s) × 40, dissociation curve analysis at 60–95 °C.	[47]
*F. subglutinans*	TCATTGGTATGTTGTCGCTCATG GTGATATGTTAGTACGAATAAAGGGAGAAC	C	95 °C for 5 min, (95 °C for 15 s, 65 °C for 60 s) × 40, dissociation curve analysis at 60–95 °C.	[47]
*F. verticillioides*	CGTTTCTGCCCTCTCCCA TGCTTGACACGTGACGATGA	C	95 °C for 5 min, (95 °C for 15 s, 65 °C for 60 s) × 40, dissociation curve analysis at 60–95 °C.	[47]
Enniatin genotype	AGCAGTCGAGTTCGTCAACAGA GGCYTTTCCTGCGAACTTG Probe: FAM-CCGTCGAGTCCTCT-MGB	B	95 °C for 20 s, (95 °C for 3 s, 60 °C for 30 s) × 40	[49]

A—2 µL gDNA, 14.3 µL H_2_O, 6.7 µM of each primer, 1.7 µM of the probe, 3.6 µL TaqMan Fast Advanced Master Mix (Applied Biosystems, Foster City, CA, USA). B—2 µL gDNA, 10.8 µL H_2_O, 6.7 µM of each primer, 1.7 µM of the probe, 7.2 µL TaqMan Fast Advanced Master Mix (Applied Biosystems, Foster City, CA, USA). C—2 µL gDNA, 8.5 µL H_2_O, 1 µM of each primer, 12.5 µL 2× SYBR Green PCR Master Mix (Applied Biosystems, Foster City, CA, USA).

## Data Availability

Assembled genomes of *Fusarium* spp. can be accessed at the NCBI bioproject under the accession number PRJNA730356.

## Supplementary Materials

The following are available online at https://www.mdpi.com/article/10.3390/toxins13120884/s1, Table S1: Identification of *Fusarium* species and enniatin genotype by PCR analyses, Table S2: Identification of *Fusarium* species using BLAST software, Figure S1: Bayesian inference phylogeny from *tef-1α* sequences of isolates from Equiseti clade, Figure S2: Bayesian inference phylogeny from *rpb1* sequences of isolates from Equiseti clade, Figure S3: Bayesian inference phylogeny from *rpb2* sequences of isolates from Equiseti clade, Figure S4: Bayesian inference phylogeny from *cam* sequences of isolates from Equiseti clade, Figure S5: Bayesian inference phylogeny from *tub2* sequences of isolates from Equiseti clade, Figure S6: Bayesian inference phylogeny from *top1* sequences of isolates from Equiseti clade, Figure S7: Bayesian inference phylogeny from *pgk* sequences of isolates from Equiseti clade, Figure S8: Bayesian inference phylogeny from *lsu* sequences of isolates from Equiseti clade.
