# Supplementary material for: Patterns of Diversity of Fusarium Fungi Contaminating Soybean Grains
Source: Toxins (Basel). 2021 Dec 10;13(12):884. doi: 10.3390/toxins13120884 (PMC8706617; doi:10.3390/toxins13120884)
Supplement: Supplementary file 1 [file toxins-13-00884-s001.zip › toxins-1416466-sm-final.pdf]

toxins

MDPI

Supplementary Materials: Patterns of Diversity of *Fusarium* Fungi Contaminating Soybean Grains

Maciej Żelechowski, Tomasz Molcan, Katarzyna Bilska, Kamil Myszczyński, Jacek Olszewski, Krzysztof Karpiesiuk, Joanna Wyrębek and Tomasz Kulik

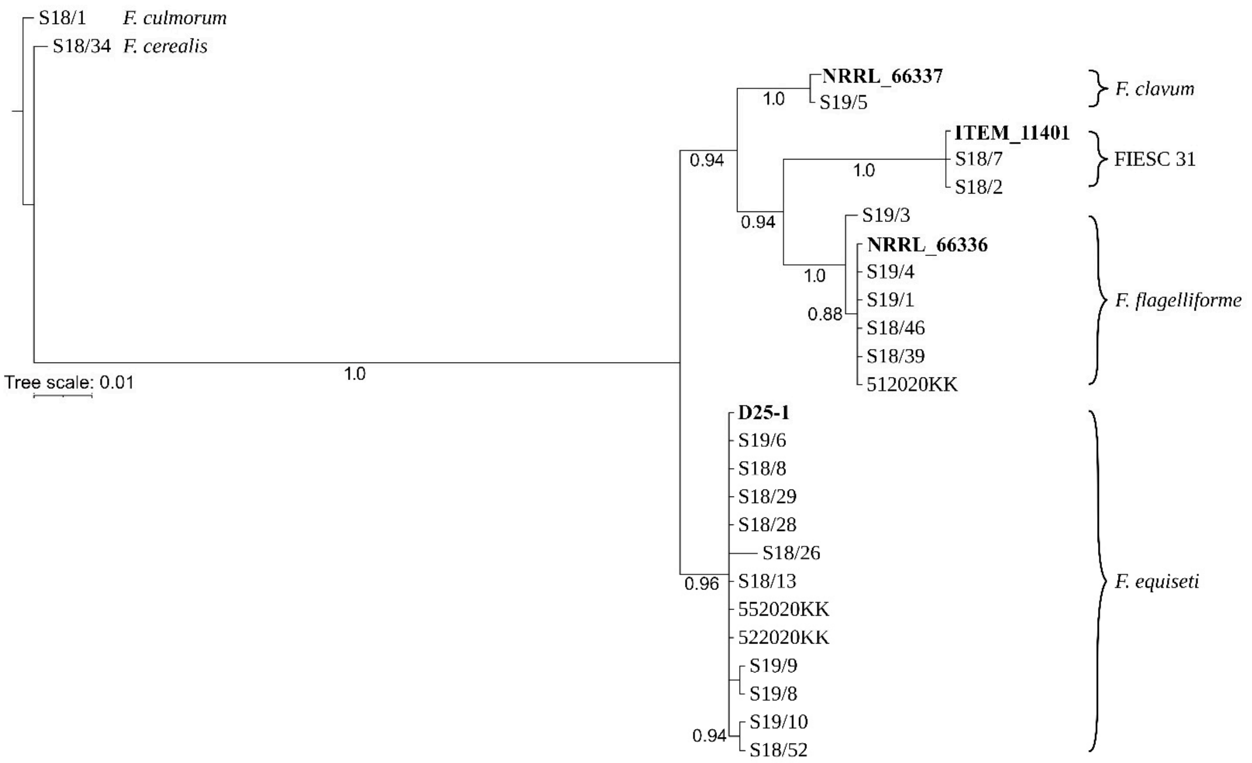

Figure S1. Bayesian inference phylogeny from *tef-1α* sequences of isolates from Equiseti clade.

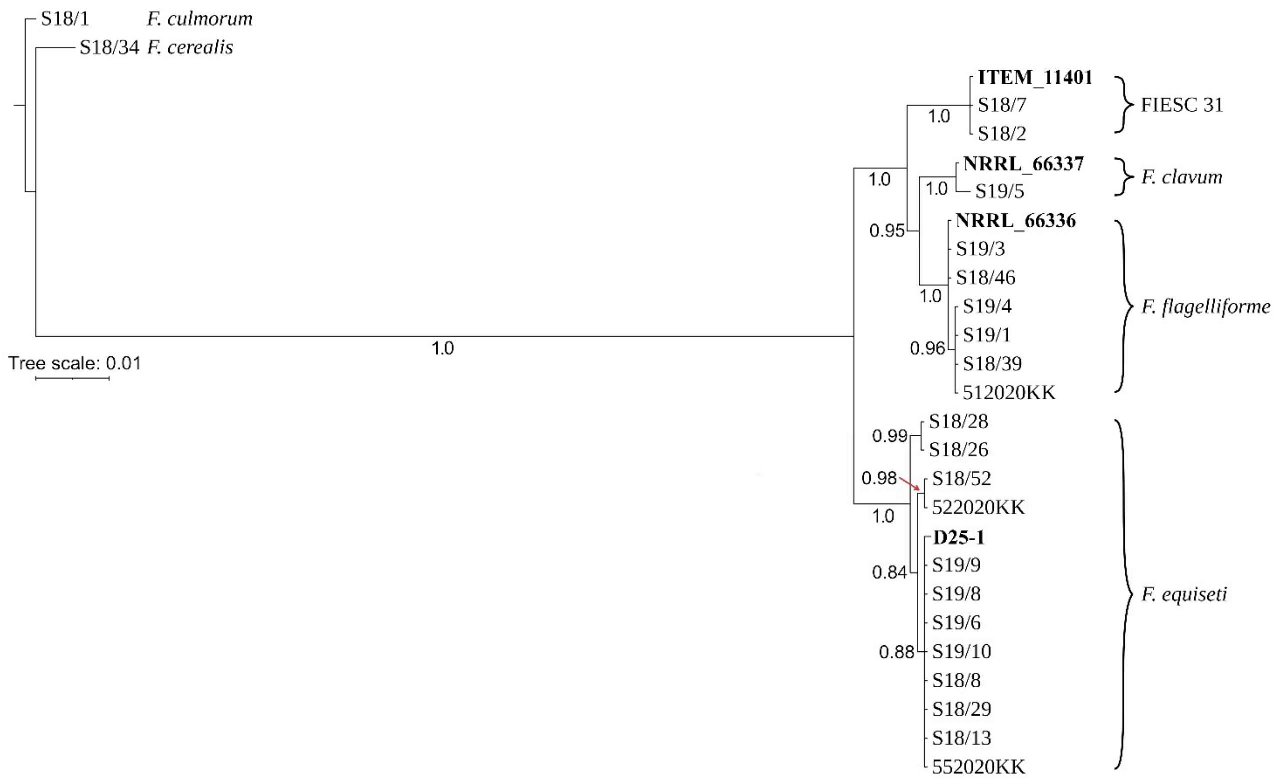

Figure S2. Bayesian inference phylogeny from *rpb1* sequences of isolates from Equiseti clade.

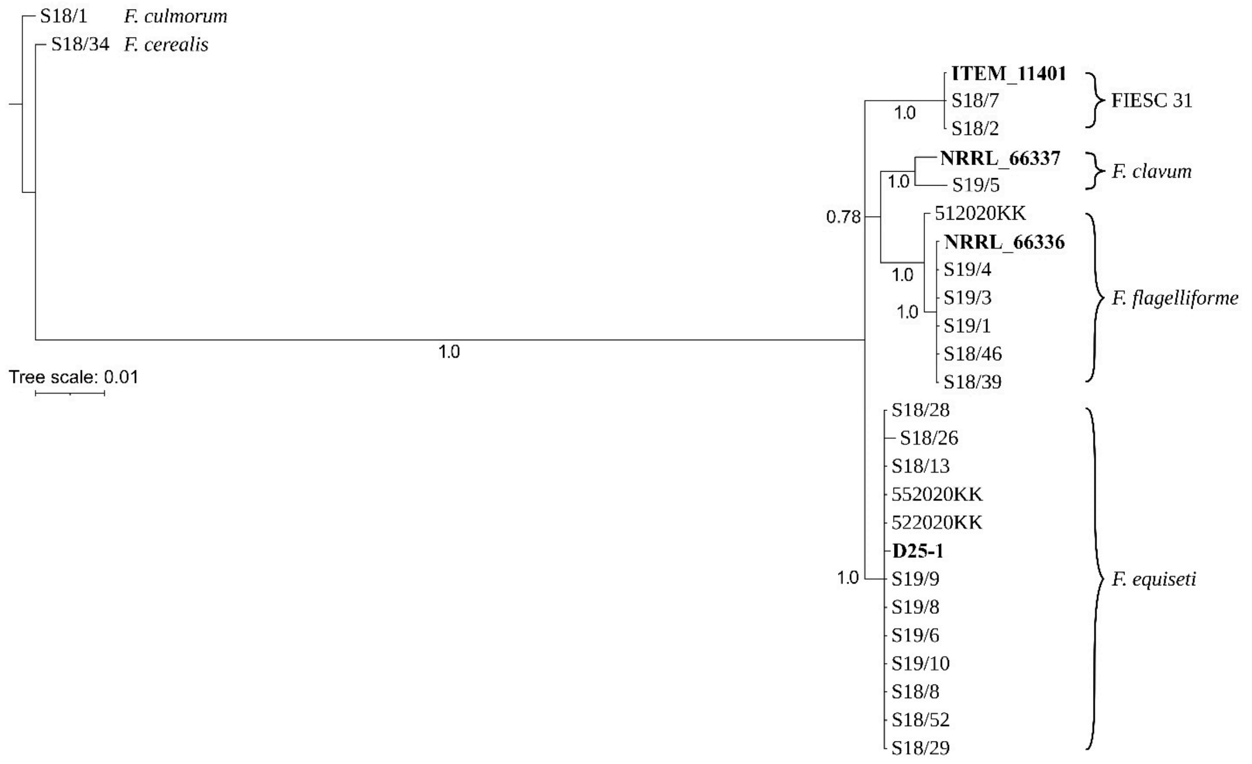

Figure S3. Bayesian inference phylogeny from *rpb2* sequences of isolates from Equiseti clade.

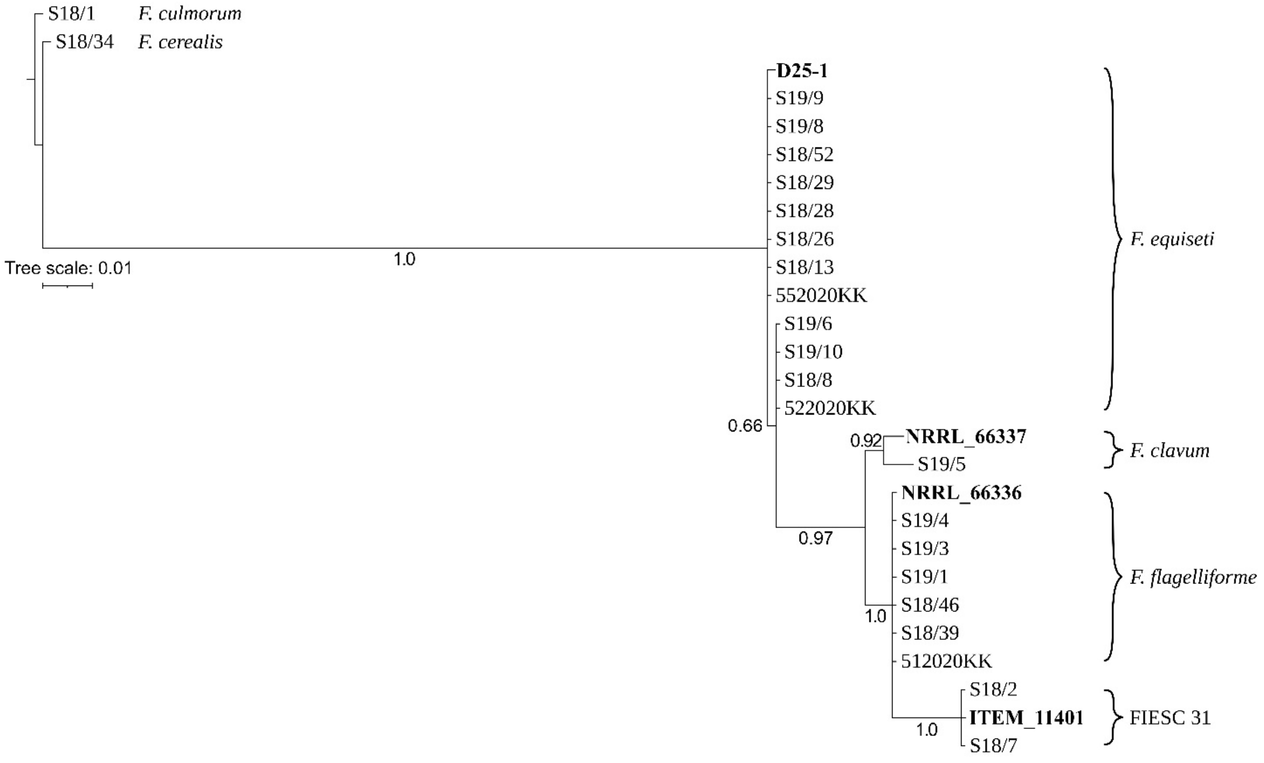

Figure S4. Bayesian inference phylogeny from *cam* sequences of isolates from Equiseti clade.

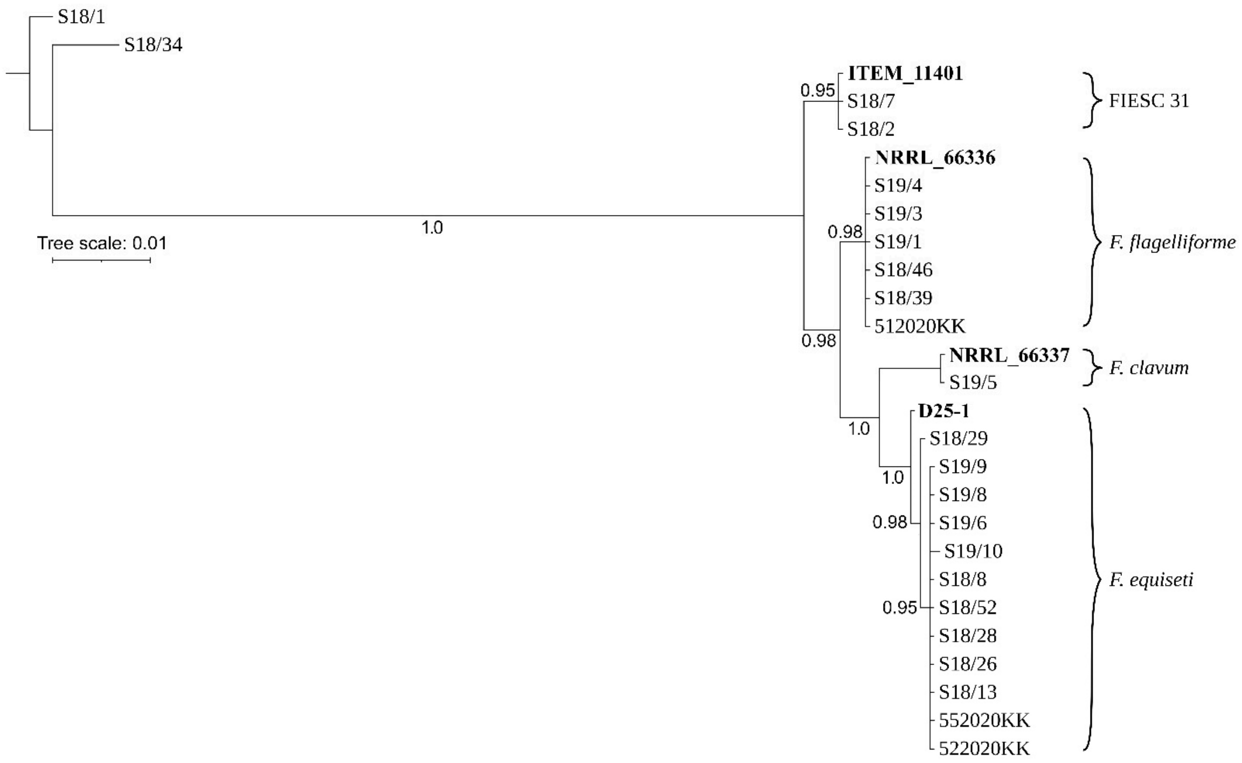

Figure S5. Bayesian inference phylogeny from *tub2* sequences of isolates from Equiseti clade.

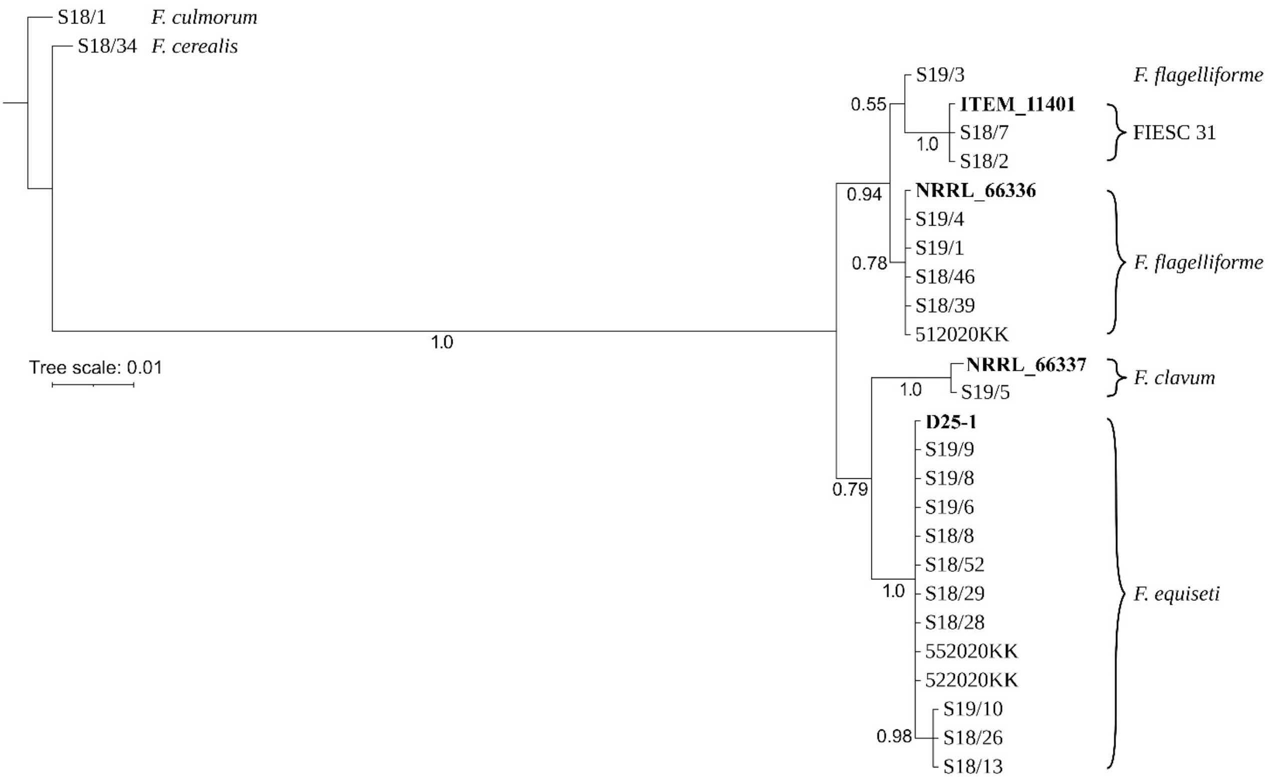

Figure S6. Bayesian inference phylogeny from *top1* sequences of isolates from Equiseti clade.

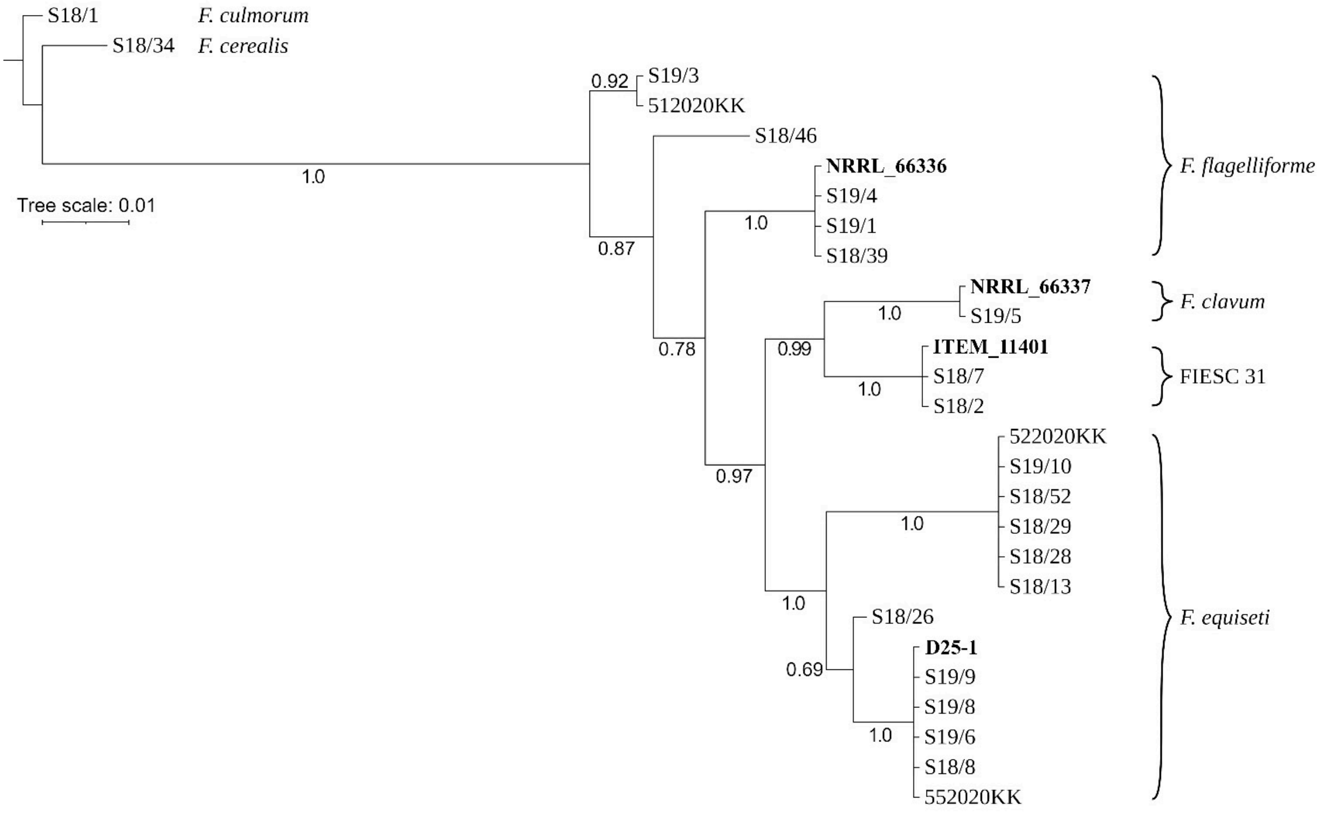

Figure S7. Bayesian inference phylogeny from *pgk* sequences of isolates from Equiseti clade.

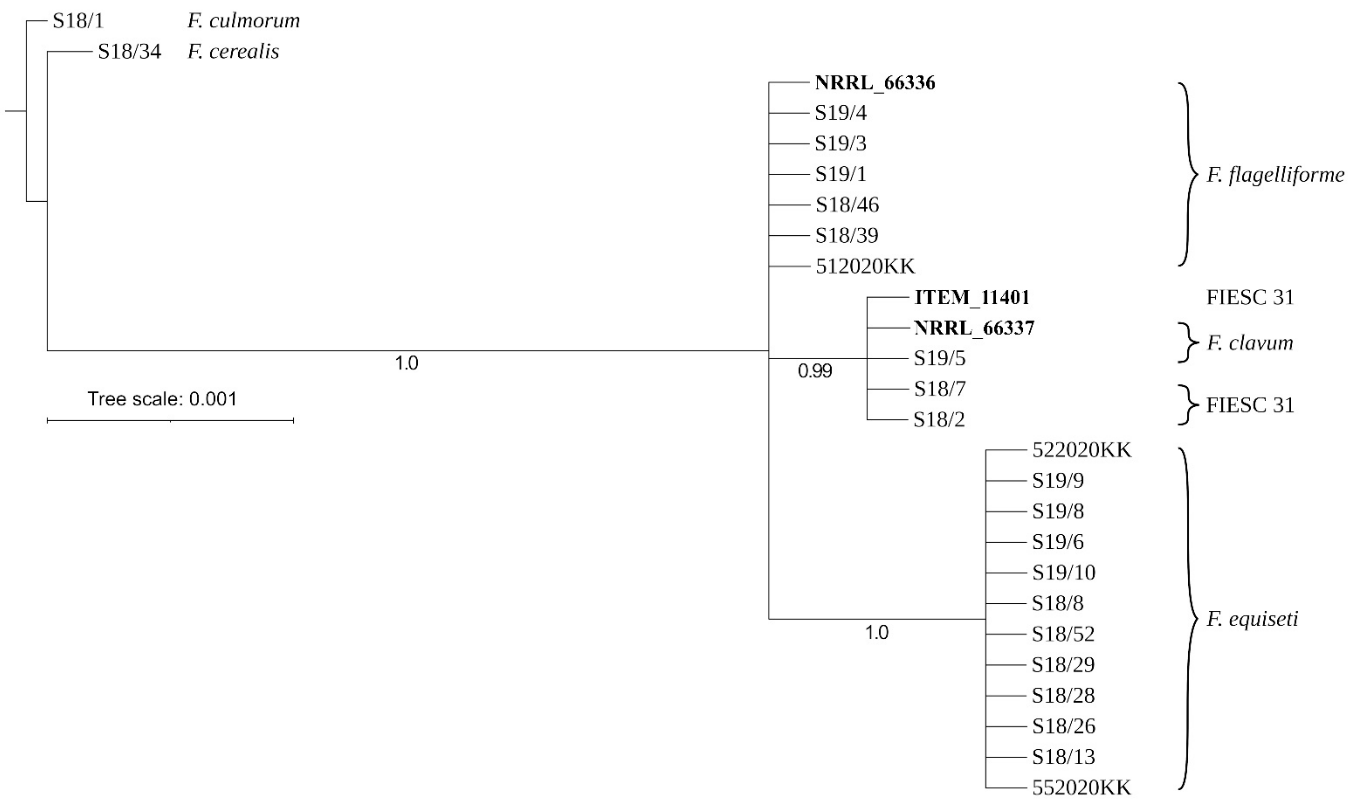

Figure S8. Bayesian inference phylogeny from *lsu* sequences of isolates from Equiseti clade.
